# Diarrhoea among Children Under Five Years of Age Residing in a Village Development Committee: A Descriptive Cross-sectional Study

**DOI:** 10.31729/jnma.7322

**Published:** 2022-05-31

**Authors:** Rimu Mishra, Alok Acharya, Amar Kumar Yadav, Rupesh Kumar Shreewastav

**Affiliations:** 1 Department of Community Medicine, Nobel Medical College Teaching Hospital, Biratnagar, Morang, Nepal; 2 Department of Biochemistry, Nobel Medical College Teaching Hospital, Biratnagar, Morang, Nepal

**Keywords:** *children*, *hygiene*, *prevalence*, *sanitation*, *water*

## Abstract

**Introduction:**

Diarrhoea is an important cause of morbidity and mortality among young children. It is reported that a large number of under five-year children still die of diarrhoea in Nepal. Nepal is striving for reducing childhood mortality by two thirds. The aim of this study is to find out the prevalence of diarrhoea among children under five years of age in a village development committee.

**Methods:**

This was a descriptive cross-sectional study performed among 356 children below 5 years of age residing in the Village Development Committee between January, 2018 and December, 2018 after getting ethical approval from the Institutional Review Committee (Reference number: 209/2017). A semi-structured questionnaire was used to collect the demographic data and other pertinent information. Convenience sampling was done. The data were entered into Microsoft Excel version 2016 and analyzed using the Statistical Package for the Social Sciences version 23.0. Point estimate at 95% Confidence Interval was calculated along with frequency and proportion for binary data and mean with standard deviation for continuous data.

**Results:**

Out of 356 children less than five years of age, diarrhoea was prevalent in 156 (43.82%) (38.6748.97 at 95% Confidence Interval). Eighty-six (55.12%) were males and 70 (44.87%) were females.

**Conclusions:**

The prevalence of diarrhoea in our study was higher when compared to similar studies conducted in similar settings.

## INTRODUCTION

The World Health Organization (WHO) estimates that over 2.2 million deaths due to diarrhoea infections occur annually, especially among children less than five years of age.^1^ WHO estimates that 88% of all diarrhoeal diseases are due to unsafe water supply, inadequate sanitation and poor hygiene practices. For the year 2002 alone, an estimated 1.1 billion people lacked access to improved water sources.^2^

Nepal is considered to be the least developed country in Asia where 80% of the population lives in the rural areas and 36% of the people practise open defecation in fields or bushes having a poor hygiene and sanitation facility leading to diarrhoea.^3,4^ It is reported that some 13,000 under 5-year children still die of diarrhoea in Nepal.^5,6^ Therefore extensive information is required for its management.

The aim of this study is to find out the prevalence of diarrhoea among children under 5 years of age residing in a village development committee.

## METHODS

A descriptive cross-sectional study was designed and conducted on children less than 5 years of age at Jhorahat, Village Development Committee (VDC) of Morang district from January, 2018 to December, 2018 after getting the ethical approval from the Institutional Review Committee (IRC) of Nobel Medical College Teaching Hospital (Reference number: 209/2017). Households with at least a single living child under 5 years of age residing in Jhorahat VDC were enrolled in the study. Children suffering from other diseases except for diarrhoea during the study period and those not willing to participate in the study were excluded. A convenience sampling was done. The sample size was calculated by using formula:

n = (Z^2^ × p × q) / e^2^

  = (1.96^2^ × 0.14 × 0.86) / 0.04^2^

  = 290

Where,

n = minimum required sample size
Z = 1.96 at 95% Confidence Interval (CI)
p= prevalence of diarrhoea, 14%^7^
q = 1-p
e = margin of error, 4%

The minimum required sample size was 290. However, a sample was of 356 children less than 5 years of age. The entire participant's guardian had signed the informed consent for the study. A diagnosis of diarrhoea was made with the passage of loose or watery stools, typically at least 3 times in a 24-hour period.^1^

A semi-structured questionnaire was used to collect the socio-demographic characteristics, knowledge, attitude, and practice on diarrhoea, drinking water source, storage and treatment, hygiene and sanitation status. The height and weight of each child were measured. Simplified field tables (Z-scores) of the WHO child growth standards chart were used to access the nutritional status of children.

The data were entered using Microsoft Excel version 2016 and analyzed using the Statistical Package for the Social Sciences version 23.0. Point estimate at 95% Confidence Interval was calculated along with frequency and proportion for binary data and mean with standard deviation for continuous data.

## RESULTS

**Figure 1:**
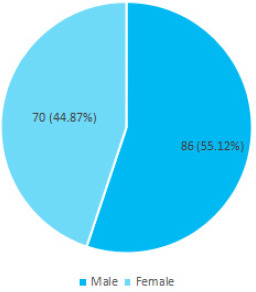
Sex wise distribution of diarrhoea in children (n= 156).

Among 356 children less than five years of age studied, the prevalence of diarrhoea was found to be 156 (43.82%) (38.67-48.97 at 95% Confidence Interval). Among 156 participants who had diarrhoea, 86 (55.12%) were males and 70 (44.87%) were females (Figure 1).

The highest number of diarrhoea cases 77 (49.04%) were from the age group 1 to 3 years, the second, being age group 3 to 5 years were 37 (40.66%), and children less than 6 months of age 17 (31.48%) had the least occurrence of diarrhoea (Table 1).

**Table 1:** Diarrhoea in different age groups (n = 156).

Age group for children	Sex	n (%)
	Males	10 (32.26)
**Less than 6 months**	Females	7 (30.43)
	Total	17 (31.48)
	Males	13 (41.94)
**6 months to 1 year**	Females	12 (52.17)
	Total	25 (46.30)
	Males	38 (44.71)
**1 year to 3 years**	Females	39 (54.17)
	Total	77 (49.04)
	Males	25 (53.19)
**3 years to 5 years**	Females	12 (27.27)
	Total	37 (40.66)

One twenty-one (77.56%) of the children with diarrhoea consumed drinking water obtained from handpumps. Only 105 (29.49%) of households used to store water for drinking purposes separately. Fifty-five (35.26%) children's households stored water for drinking purposes separately. One hundred twenty-six (80.77%) children's households used to cover the stored drinking water container with a lid. Only 72 (20.2%) of household of these children practised direct pouring with hands not dipping in water. One hundred thirty-five (47.5%) children from families practised hand dipping (Table 2).

**Table 2:** Diarrhoea by source, separate storage, covering lid and drawing of drinking water (n= 156).

	n (%)
**Water source**	
Piped water	35 (22.44)
Handpump	121 (77.56)
**Separate storage**	
Yes	55 (35.26)
No	101 (64.74)
**Covering with lid**	
Yes	126 (80.77)
No	30 (19.23)
**Hand dipping**	
No	21 (13.46)
Yes	135 (86.54)

One hundred twenty-seven (81.41%) of children practised handwashing with soap water whereas 29 (18.59%) used only plain water, mud and ash. One hundred eighteen (75.64%) of children used improved latrine facilities of water sealed kind, 26 (16.67%) practised defecating in pit latrines and 12 (7.69%) had no toilet facility in-home and used to defecate in the open area. One hundred thirteen (72.44%) children's families didn't treat their household wastes and used to throw haphazardly (Table 3).

**Table 3:** Diarrhoea by handwashing practices, toilet facilities and waste management (n = 156).

Variables	n (%)
**Hand wash**	
With soap	127 (81.41)
Without soap	29 (18.59)
**Toilet**	
Water sealed	118 (75.64)
Pit latrine	26 (16.67)
Open area/No facility	12 (7.69)
**Waste treatment**	
Yes	43 (27.56)
No	113 (72.44)

Thirteen (8.33%) children with diarrhoea were not breastfed whereas 143 (91.67%) children were breastfed (Table 4).

**Table 4:** Diarrhoea with breastfeeding and age group of children (n = 156).

Age group of children	Breastfeeding	Diarrhoea n (%)
**Less than 6 months**	Yes	15 (9.62)
	No	2 (1.28)
**6 months - 1 year**	Yes	21 (13.46)
	No	4 (2.56)
**1 - 3 years**	Yes	74 (47.44)
	No	3 (1.93)
**3 - 5 years**	Yes	33 (21.15)
	No	4 (2.56)

## DISCUSSION

The study was carried out in the Jhorahat area with the aim of finding the prevalence of diarrhoea in under-five children in this community. The prevalence of diarrhoea was found to be 43.82% in the present study. This was much higher than what was reported in the Annual Report of Nepal 2011 which was 14%.^7^ This difference in prevalence is probably due to the fact that the annual report included episodes reported at health facilities. The present study was community-based. Diarrhoea cases may not be reported to health facilities also. The prevalence of diarrhoea was highest in the 1 to 3 years age group (49%) followed by children between the age of 6 months to 1 year where the prevalence was 46.3% in this study. More episodes of diarrhoea were seen in males in all age groups except for age groups 1-3 years. The study from the slums of Tansen, Palpa^8^ found the prevalence of under-five to be 48% which is similar to the present study. In Southern Nepal, the prevalence of under-five diarrhoea was reported to be 36.6%.^9^ The prevalence of diarrhoea is still very high in children under 5 years of age. Similar to our findings, a study done in Dhulikhel found a higher incidence of diarrhoea in children below 24 months (2 years) of age.^10^ However, another study in Delhi found the highest occurrence of diarrhoea among children in the 7-12 months age group.^11^

Families where the water was covered with a lid the prevalence of diarrhoea was found to be 126 (49.8%) and in families where it was not covered, it was found to be 30 (29.1%). In the study done in Malawi in which the improved bucket for storing drinking water had a reduction of diarrhoea among children under 5 by 31%.^12^ Also, the study done in Bolivia whereby proper storage exhibited less *Escherichia coli* contamination and families in the intervention group had 43% fewer diarrhoea than in the control group.^12^

Two hundred eighty-eight (80.9%) families of the children used soap water whereas 68 (19.1%) didn't use soap for washing hands. It is evident that children tend to put their own fingers, hands and various objects inside their mouth frequently leading to feco-oral contamination followed by diarrhoea. Handwashing behaviour of family alone is not protective against diarrhoea in children so their activities and hygiene should be carefully observed and caretakers should wash the hands of their children frequently. Various studies found proper handwashing lowers diarrhoeal incidence.^8,13-15^ In another study done in Sarlahi, Nepal it was found that effective handwashing was related to a statistically significant lower mortality rate among neonates.^16^

Two hundred eighty-six (80.3%) families had improved latrine facilities, 51 (14.3%) houses had pit latrines and 19 (5.3%) had no toilet facilities. But there was a difference in the prevalence of diarrhoea in children where facilities of a toilet with water sealed 118 (41.3%), pit latrine 26 (51.0%) or open area 12 (63.2%) was used. The prevalence was higher in families which used open areas or pit latrines as compared to families which used water-sealed. A study done in Dhulikhel showed that 77% of families had well-maintained toilets in their house and 23% practised open defecation or used public toilets.^10^ Presence of proper toilet facilities was also associated with a lower incidence of childhood diarrhoea.^8^ Highest occurrence of diarrhoea was noted in families who hadn't any toilet facilities in-home and practised open defecation.^11^

Two hundred thirty-six (66.3%) families didn't practice treating the household waste properly and were throwing it in the surroundings. The remaining 120 (33.7%) were treating household waste either by burning, burying or preparing compost. There was a lower prevalence of diarrhoea in families who used to treat household waste 43 (35.8%) as compared to families who didn't treat 113 (47.9%). Other studies showed poor hygienic practices^17^ and the presence of garbage near home^18^ were significant risk factors for childhood diarrhoea.

A total of 338 (95%) children were adequately breastfed. Breastfeeding practices were studied with the prevalence of diarrhoea in under-five children was studied. There were 54 children below 6 months of age and 52 (92.3%) of them were exclusively breastfed. The prevalence of diarrhoea in children below 6 months of age was 17 (31.5%). The number of children in the age group 6 months to 1 year was 54. Forty-nine (90.7%) children in this group were still being breastfed along with other supplementary feeds. The prevalence of diarrhoea in breastfed children was 21 (42.3%) In a study done in southern Nepal, it was found that partial breastfeeding was associated with higher odds of childhood diarrhoea.^9^ Other studies done in Palpa,^8^ Delhi,^11^ Zimbabwe^18^ revealed less prevalence of diarrhoea in children who were adequately breastfed.

There are some limitations of the present study. The cases of diarrhoea had been included from one VDC. It would have been interpreted more accurately if more cases of diarrhoea would have been taken from other regions of the Morang district.

## CONCLUSIONS

The prevalence of diarrhoea among children from our study was found to be higher when compared to similar studies done in the country. The findings of this study have revealed that diarrhoea was very common among children less than five years of age in Jhorahat. It was also noted that the cases of diarrhoea were directly proportional to breastfeeding, source of water, hand dipping while drawing water from the storage container and household waste management.
